# Patè Olive Cake: Possible Exploitation of a By-Product for Food Applications

**DOI:** 10.3389/fnut.2019.00003

**Published:** 2019-02-05

**Authors:** Maria Tufariello, Miriana Durante, Gianluca Veneziani, Agnese Taticchi, Maurizio Servili, Gianluca Bleve, Giovanni Mita

**Affiliations:** ^1^Consiglio Nazionale delle Ricerche—Istituto di Scienze delle Produzioni Alimentari, Lecce, Italy; ^2^Dipartimento di Scienze Agrarie, Alimentari e Ambientali, Università degli Studi di Perugia, Perugia, Italy

**Keywords:** bioactive compounds, fermentation, functional product, patè olive cake, starter

## Abstract

Patè Olive Cake (POC) is a new by-product derived from recently introduced new decanters in the olive oil production process. POC, is essentially composed of water, olive pulp and olive skin, and is rich in several valuable bioactive compounds. Moreover, it still contains about 8–12% residual olive oil. We characterized the main bioactive compounds in POC from black olives (cv. *Leccino* and *Cellina di Nardò*) and also verified the biotechnological aptitude of selected yeast and lactic acid bacteria from different sources, in transforming POC into a new fermented product. The strategy of sequential inoculum of *Saccharomyces cerevisiae* and *Leuconostoc mesenteroides* was successful in driving the fermentation process. In fermented POC total levels of phenols were slightly reduced when compared with a non-fermented sample nevertheless the content of the antioxidant hydroxytyrosol showed increased results. The total levels of triterpenic acids, carotenoids, and tocochromanols results were almost unchanged among the samples. Sensory notes were significantly improved after fermentation due to the increase of superior alcohols, esters, and acids. The results reported indicate a possible valorisation of this by-product for the preparation of food products enriched in valuable healthy compounds.

## Introduction

The olive oil industry has a great global importance and economic impact especially for Mediterranean countries, which contribute to about 92% of the world's olive oil production (about 3 million tons seasons 2017–2018). EU contributes 66% to global olive oil production and Spain, Italy and Greece are the main EU producers contributing to 38, 13, and 10%, respectively (1). The high quantity of olives processed by the milling industry results also in the production of a large amount of various by-products that must be properly managed to limit serious environmental impact. For these reasons at the present time these by-products represent a relevant cost for the milling industry. In particular the two-phase milling process produces a large amount of olive pomace also called olive cake that contains olive pulp, skin, stone, and water and is known to be a phytotoxic and environmental pollutant. Nevertheless, it is also known that olive pomace is a rich source of valuable compounds that can be recovered (2, 3). Olive drupes are indeed very rich in phenolic compounds although most of these compounds (about 98%) are lost in the olive mill by-products (4–6).

The health properties as well as the biological activities of these compounds, particularly polyphenols have been highlighted in several studies (7–9). Many reports are available indicating the protective effects of polyphenols toward atherosclerosis, diabetes, obesity, and many other chronic diseases (10, 11). Moreover, the European Food Safety Authority stated “*Olive oil polyphenols contribute to the protection of blood lipids from oxidative stress”* (12).

Due to the presence of high valuable compounds, olive mill by-products have attracted a great deal of attention for long time and many attempts have been made to exploit them in various industrial sectors including the feed and food industries (13, 14). Phenolic compounds, pectic polysaccharides and fibers of lignocellulose from olive by-products have been proposed for potential applications as a source of oligo-, di-, and mono-saccharides, as antioxidants, in the development of gelling and stabilizing agents, food packaging and preservation and food fortification.

In particular, olive pomace extracts were added to soy oil for fried potatoes enrichment of several bioactive compounds (15, 16) and for the enrichment of nutritional traits of fermented milk (17).

However, up to now, real successful and commercial production has been very limited. One of the main drawbacks in the use of olive pomace for food and feed applications is the presence of lignin (resulting from crushed stones) that contributes to the poor digestibility. De-stoned olive cake added into a concentrate-based diet for lambs resulted in an improvement of the oxidative stability and the nutritional quality of meat by increasing vitamin E content in muscle (18).

Recently a new technology for olive oil extraction has been proposed that adopts an innovative two-phase decanter (Leopard, Pieralisi, Jesi, Italy). This decanter, besides producing a dehydrated husk, also produces a novel by-product named “patè” or “patè olive cake” (POC) consisting of a semisolid de-stoned olive cake that includes olive pulp, olive skin, olive mill wastewater while it is very poor in lignin. Due to the presence of residual olive oil (8–12%) POC contains fatty acids (palmitic acid, oleic acid, and polyunsaturated fatty acids). Moreover, it contains triterpenic acids (mainly oleanolic acid and maslinic acid) and several phenolics including hydroxytyrosol, tyrosol, secoiridoids derivatives, verbascoside, and many phenolic acids (19, 20). POC also contains carotenoids and lignans. Consequently POC has been suggested for various food and feed applications. Recently Padalino et al. (20) developed a spaghetti enriched with 10% POC which resulted in an increased total of polyphenol content and also showed a PUFA/SFA ratio higher than the control. Moreover, Cecchi et al. (21) reported anti-aging effects of POC on human fibroblast cells, while the preparation of olive pomace-based polyphenol rich extracts encapsulated in cyclodextrins to be used as food antioxidants has been successfully tested by Cepo et al. (22).

Starter driven fermentation has been extensively studied for table olives (23–28), their derived products (29) and for the bioremediation of olive mill wastewaters (30–36). At present, no applications of this approach have been developed for POC.

In this paper, for the first time, a production method of fermented POC, obtained from *Cellina di Nardò* and *Leccino* olives, using the Leopard decanter during the production of extra virgin olive oil, was studied. For this purpose, key steps for the selection of microorganisms as possible starters for POC fermentation were determined. Process parameters and the protocol of a sequential inoculum approach using, firstly, a selected strain of *Saccharomyces cerevisiae* and then a selected strain of *Leuconostoc mesenteroides* were adopted. Chemical and microbiological characterizations of the fermented products were carried out.

## Materials and Methods

### Patè Olive Cake Samples (POC)

Olives (cv *Cellina di Nardò* and *Leccino)* were harvested from organic orchards in Salento Area (South Apulia Region, Italy) during seasons 2014–2015, 2015–2016, and 2016–2017. Five hundred kilo gram olives for each cultivar were manually harvested at a maturity index of about 3–4 (skin turning black or fully black). Olives were immediately transported to the mill, washed twice and selected by caliber and color. Olive were then milled using a Multi-Phase Decanter technology (Leopard, Pieralisi, Jesi, Italy). Aliquots of Patè Olive cake (POC) were sterilized at 121°C for 4 min to reduce/block possible spontaneous fermentations without affecting matrix composition.

Yeast strains used in this study were: a wine yeast *Saccharomyces cerevisiae* strain ENARTIS FERM SC (ESSECO, Trecate, Italy) (WSC), two brew yeasts [Australian bitter and pale ale malt (YPA), Coopers, Australia] (YAB), a commercial baker's yeast (BY), *Candida boidinii* ISPA-LE-A5y (A5y) isolated from olive mill wastewaters, *Saccharomyces cerevisiae* ISPA-LE-KI 30-1 (KI-30-1) isolated from fermented table olives, *S. cerevisiae* ISPA-LE-LI 60-17 (LI-60-17) from fermented table olives. Lactic acid bacteria strains used in this study were: *Lactobacillus plantarum* ISPA-LE-C-11 (C-11), *L. plantarum* ISPA-LE-C-34 (C-34), *Leuconostoc mesenteroides* ISPA-LE-K-1 (K-1), *L. mesenteroides* ISPA-LE-B-T3-35 (BC-T3-35). All these bacterial strains were previously isolated from fermented table olives.

### Microbiological Analyses

Microorganisms present in fermented POC samples were analyzed by serial dilutions with 0.1% (w/v) peptone water. After dilution, samples were applied to agar plates containing: Man, Rogosa, and Sharpe Agar (MRS, LABM, UK) for LAB isolation and Plate count Agar (PCA, LABM, UK) for total bacterial count in presence of 0.05 g/L of nystatin and incubated at 30°C for 48–72 h; Violet Red Bile Glucose Agar (VRBGA, LABM, UK) for *Enterobacteriaceae* identification (37°C for 18–24 h); Baird Parker Agar Base (Oxoid) for the enumeration of coagulase positive Staphylococci incubated at 37°C for 24–48 h; Violet Red Bile Agar (VRBA, Oxoid) for the detection and enumeration of coli-aerogenes bacteria incubated at 37°C for 24–48 h; Mannitol Salt Agar (MSA, Oxoid) for the isolation of pathogenic Staphylococci incubated at 37°C for 18–72 h; Sulphite-Polymyxin-Sulphadiazine Agar (SPS, Oxoid) for the detection of *Clostridium perfringens* incubated at 35–37°C for 18–48 h under anaerobic conditions; Sabouraud Dextrose Agar (LABM, UK) (yeasts identification) in presence of 0.05 g/L of kanamycin and 0.1 g/L of ampicillin by incubation for 2–4 days at 25°C.

### LAB and Yeast Selection

As a first step of this work, the highest amount of lyophilized POC in growth medium that can be tolerated by yeast and LAB strains without affecting their growth was determined. To this scope, YPD and MRS media were supplemented with different amounts (0, 10, 25, 50, 75, 80, 90, 100% w/v) of lyophilized POC. The pH of the media was adjusted to 4–4.5 before thermal treatment of 10 min at 110°C. Yeasts and LAB were grown on YPD and MRS broth, respectively and adjusted to 10^7^ CFU mL^−1^ final concentration (corresponding 600 nm of ≈ 1.0 optical density). Fifteen microliters of each yeast and LAB inoculum were spotted onto YPD and MRS agar media containing the different amounts of POC. The agar plates were incubated for 5–7 days at 28°C. Each assay was carried out in triplicate. Each experiment was performed twice.

In the second step, laboratory-scale fermentations were carried out for the selection of microbial starters able to ferment POC. Yeasts and LAB strains were firstly grown as a pre-adaptation period in ½ strength YPD (yeast extract 0.5% w/v, peptone 1% w/v, glucose 1% w/v) and half strength MRS (27.6 g L^−1^, VWR, Belgium), respectively, containing 25% w/v POC and subsequently inoculated at a concentration of 5 × 10^6^-10^7^ CFU mL^−1^ in a liquid medium containing half strength YPD for yeasts, and a liquid medium containing half strength MRS for LAB isolates, both containing 50% w/v OPC. Fermentations were performed using 500 ml glass bottles with 250 ml of YPD-POC and MRS-POC at 28°C for 60 days. The fermentative activities of each yeast and LAB strains were followed by monitoring consumption of main sugars, evolution of most important organic acids, phenols and volatile compounds.

### Pilot-Scale Fermentation

Fermentations were performed in plastic vessels (10 kg capacity). Sterilized POC samples of *Cellina di Nardò* and *Leccino* were diluted 1:1 with distilled water. After addition of 0.5% (w/v) yeast extract, 0.5% (w/v) peptone, 0.5% (w/v) glucose, vessels were maintained at ambient temperature (18–22°C).

Starter cultures were inoculated by a sequential inoculation strategy: *Cellina di Nardò* and *Leccino* POC samples were first inoculated with *Saccharomyces cerevisiae* ISPA-LE-KI 30-1 and then (after 30 days) with *Leuconostoc mesenteroides* ISPA-LE-B-T3-35.

About 10^7^ CFU/ml yeast and LAB starter strains were used to inoculate, OPC samples. Yeasts starter firstly drove the fermentation process. Throughout the fermentation pH, sugars consumption and organic acids production were monitored. At the end of the yeast fermentations LAB were added. This time point was determined by the appearance of specific compounds (higher alcohols, terpenes, etc.), the whole process was considered terminated when compounds such as esters and acetate esters were detected (23, 24).

### Detection and Quantification of Sugars, Organic Acids, and Alcohols

The soluble sugars were extracted from dried olive paste samples according to Eris et al. (37) with some adjustments. Two mililiter of ethanol: water (80:20 v/v) were added to POC powders (0.05 g). The mix, after stirring, was incubated at 85°C for 1 h in a water bath. The ethanolic phases were collected and evaporated until dry at 55°C. Organic acids were determined according to Ergönül and Nergiz (38), using a mixture of water: methanol solution (75:25 vol/vol) as solvent. The supernatant was recovered by centrifugation at 5,000 × g for 10 min. This extraction was repeated three times and the collected supernatants were evaporated until dry.

### Polyphenols Extraction and Analysis

The polyphenol extraction was carried out as reported by Servili et al. (39) with minor changes. Five grams of each sample were homogenized with 50 mL of methanol (80%, v/v) containing 20 mg/L of butylated hydroxytoluene. The methanol was evaporated and the aqueous extract was used for solid-phase extraction (SPE) of phenols. The SPE procedure was performed by loading 1 mL of the aqueous extract into a 1,000 mg Bond Elut Jr-C18 cartridge (Agilent Technologies, USA), using 50 mL of methanol as the eluting solvent. The solvent was evaporated under vacuum at 30°C, the residue was dissolved in 1 mL of methanol. Quali-quantitative analysis of polyphenols was carried out as described by Servili et al. (39) using an HPLC system 1100 Series (Agilent Technologies, California, USA) equipped with a A C18 Spherisorb column ODS-1 250 x 4.6 mm with a particle size of 5 μm (Waters, Milford, MA, USA). Lignans [(+)-pinoresinol] were detected by using the FLD at an excitation wavelength of 280 nm and emission at 339 nm, whereas the other compounds were detected by using the DAD with a wavelength of 278 nm and 360 nm for rutin. The tyrosol (*p*-HPEA), vanillic acid, *p*-coumaric acid and rutin were purchased from Fluka (Milan, Italy), while the hydroxytyrosol (3,4-DHPEA) was obtained from Cabru s.a.s. (Arcore, Milan, Italy) (Arcore, Milan, Italy). Verbascoside was purchased from Extrasynthese (France). Oleacein, oleocanthal and (+)-pinoresinol were obtained from PhytoLab GmbH & Co. (Germany). Hydroxytyrosol acetate and isoverbascoside were quantified using the response factors of hydroxytyrosol and verbascoside, respectively.

### Volatile Compound Extraction From POC

Volatile compounds were extracted according to Bleve et al. (24) HS-SPME analyses were carried out using a gas chromatograph (Agilent 6890N) coupled to a mass spectrometer (5973, Agilent Technologies, USA). The Separation, identification and quantification of compounds were performed according to Bleve et al. (24).

### Triterpenic Acids Extraction and Analysis

Triplicate aliquots (1 g) of each sample were extracted with 4 mL of methanol/ethanol (1:1, v /v) in a Labsonic LBS1-10 ultrasonic bath [Falc Instruments, Treviglio (Bg), Italy] at room temperature for 1 min and centrifuged at 4,000 × g for 10 min, this procedure was repeated for six times (40). The extracts were combined and evaporated to dryness, and the residues were re-dissolved in 1 mL of methanol. Triterpenic acids were analyzed according to Durante et al. (40) using an 1,100 Series HPLC system (Agilent Technologies, California, USA) equipped with a Luna column (5 μm, 250 × 4.6 mm) (Phenomenex, Torrance, CA, USA). Samples were filtered before analysis through a 0.45 μm syringe filter (Millipore Corporation, Billerica, MA, USA).

### Isoprenoids Extraction and Analysis

Isoprenoids (tocochromanols and carotenoids) were extracted essentially as reported by Padalino et al. (20). Triplicate aliquots (1 g) of each sample was incubated with 5 mL acetone containing 0.05% (w/v) butylated hydroxytoluene (BHT), 2 mL ethanol (95% v/v), 1 mL sodium chloride (1% w/v) and 2 mL potassium hydroxide (60% w/v) at 60°C for 30 min. After cooling, 15 mL sodium chloride (1% w/v) was added. Samples were then extracted twice with 15 mL n-hexane/ethyl acetate (9:1 v/v), and the upper phases were collected, dried under nitrogen flux, dissolved in 1 mL ethyl acetate, filtered through a 0.45 μm syringe filter (Millipore Corporation, Billerica, MA, USA) and assayed by HPLC according to Durante et al. (41). Absorbance was recorded by DAD at 474 nm and 290 nm for carotenoids and tocochromanols, respectively. Peaks were identified and quantified by comparison to authentic standards.

### Lipid Extraction

Triplicate aliquots (1 g) of each sample were mixed with 5 mL of n-hexane and stirred with a mechanical stirrer (300 rpm) at 4°C for 16 h. Samples were centrifuged at 6,000 × g for 5 min. The organic phase was evaporated until dry and stored at −20°C until analysis.

### Fatty Acids Analysis

Fatty acids were derivatized according to Durante et al. (42). Dried extracts were solubilized with 3 mL KOH methanolic solution (0.5 M). Samples were incubated at 100°C for 5 min. After cooling, 2 mL boron trifluoride in methanol (12% w/v) were added, and samples were incubated for 30 min at 100°C in a water bath and then cooled in an ice bath before the addition of 1 mL of *n*-hexane and 1 mL sodium chloride (0.6% w/v). Samples were shaken (30 s) on a vortex-stirring for 5 min and centrifuged at 600 x g for 10 min. The organic upper phase was collected and 3 μL aliquots were directly injected into GC-MS as described by Durante et al. (42) using an Agilent 5977E GC/MS system equipped with a DB-WAX column (60 m, 0.25 mm i.d., 0.25 mm film thickness, Agilent).

### Statistical Analysis

The data are shown as mean values of replicate measurements (*n* = 3) with standard deviation. Student's *t*-test was applied to the polyphenols, triterpenic acids, isoprenoids, and fatty acids and a one-way analysis of variance (ANOVA) and the Tukey HSD *post hoc* test were applied to the volatile compounds to establish significant differences between means (*p* < 0.05). All statistical comparisons were performed using SigmaStat version 11.0 software (Systat Software Inc., Chicago, IL).

Principal component analysis (PCA) was used to compare possible correlated variables, such as volatile and phenolic compound content, of the POC samples during fermentations. All statistical analyses were carried out using the STATISTICA 7.0 software (StatSoft software package, Tulsa, OK, USA).

## Results

### Selection of Yeasts and LAB Candidates as Starters

In the first step selection, all yeast and LAB isolates, inoculated in YPD and MRS agar media added with different amounts (0, 10, 25, 50, 75, 80, 90, 100% w/v) of lyophilized and sterilized POC, showed the ability to grow in a way similar to the controls when a concentration ≤ 50% (w/v) POC was used (data not shown).

In order to identify yeast and LAB strains as candidate starter for POC fermentation, lab-scale fermentations in media containing 50% (w/v) POC were set up. Metabolic activities of candidate starters were followed by monitoring some key chemical parameters, such as sugars (glucose and fructose), alcohols (ethanol), phenols, organic acids, and volatiles compounds associated with the microbial metabolic activities. All yeast and LAB strains were able to almost completely consume sugars. At the end of fermentation (60–days), the glucose initial content (16.51 ± 0.41 mg/g), was greatly reduced (up to 0.6–5.7% of the initial content) in POC samples inoculated with yeast as well as in POC samples inoculated with LAB isolates (up to 0–12% of the initial content). Concerning fructose, the initial concentration, corresponding to 7.21 ± 0.41 mg/g in both media, was reduced to 0–7.2% initial content in samples containing yeast isolates and to 0–9.8% in samples inoculated with LAB isolates. No substantial differences were observed among samples fermented by yeast and LAB isolates in relation to citric, tartaric, malic, lactic, and acetic acids.

The consumption of sugars and the corresponding increase of organic acid levels revealed the progress of fermentation in all POC samples inoculated with starters.

It is worth noting that, after fermentation, the levels of the important antioxidant compound hydroxytyrosol increased in all POC samples.

### Analysis of Volatile Compounds in POC Fermented by Yeasts and LAB

**Figure 1:**
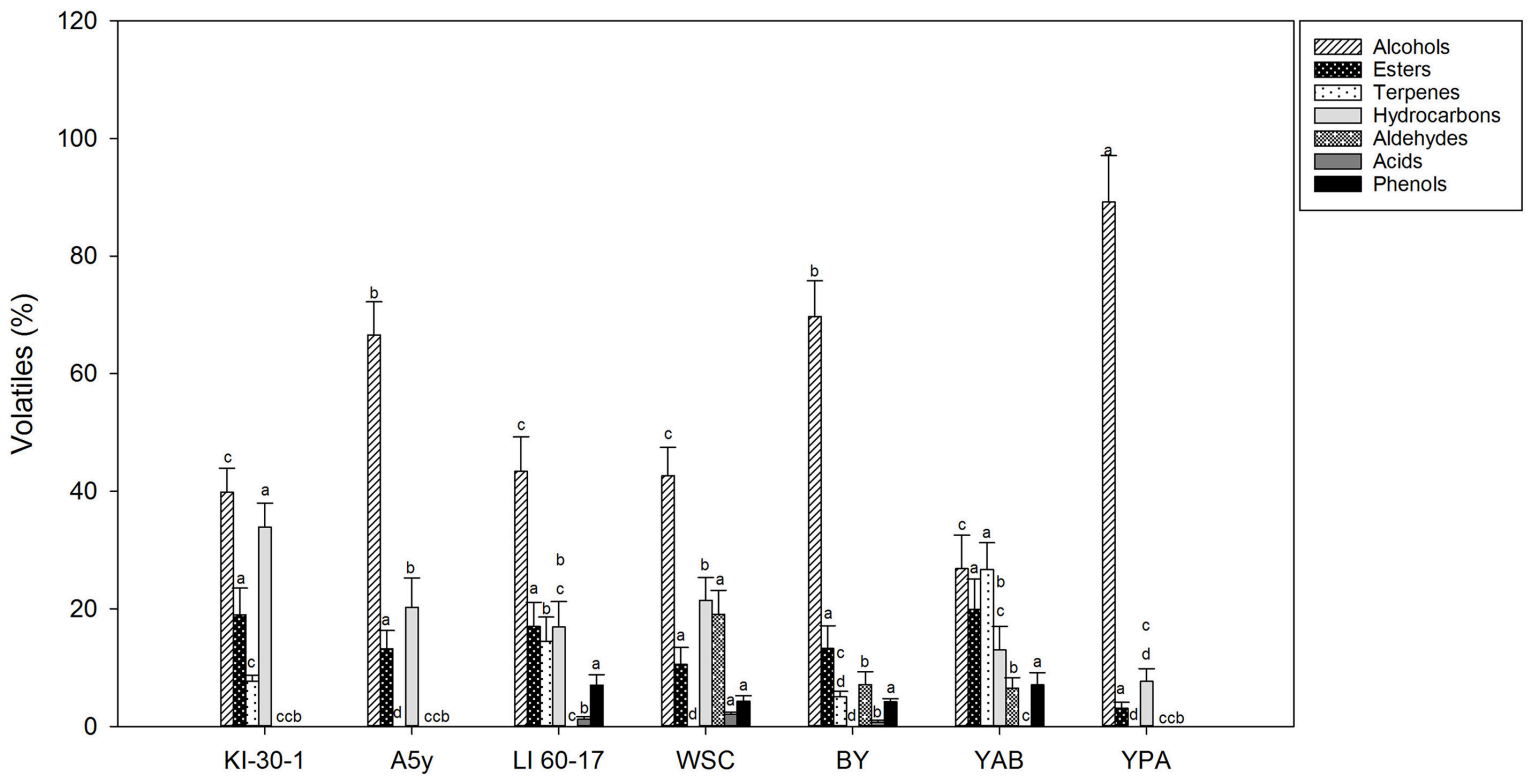
Volatile compound classes associated to YPD-POC fermented by different yeast isolates. Different letters indicate significant differences among stage of fermentation in the same volatile classes (*p* < 0.05).

**Figure 2:**
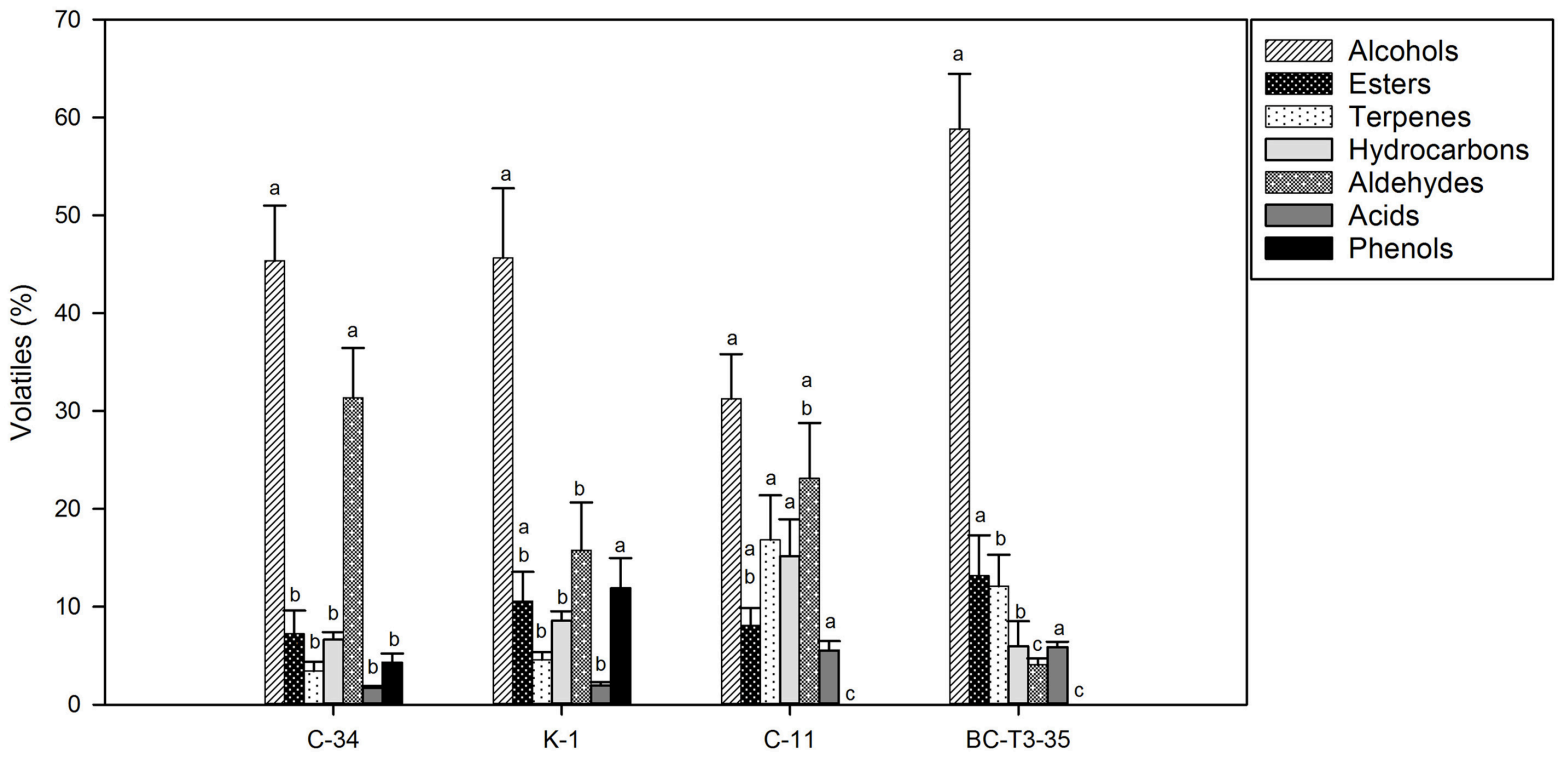
Volatile compound classes associated with MRS-POC fermented by different LAB isolates. Different letters indicate significant variations depending on the fermentation stage in the same volatile classes (*p* < 0.05).

In Figures 1, 2 concentrations (expressed in %) of volatile compounds identified in POC fermented by different yeast and LAB strains, respectively, were reported. The chemical classes of volatiles identified, were: alcohols, esters, terpenes, hydrocarbons, aldehydes, volatile acids, and volatile phenols.

Fermentation process, performed by different yeast and LAB strains, produced significant differences in terms of volatile profiles in POC samples.

Among POC samples fermented by yeasts (Figure 1 and Table S1), as expected, alcohols represented the most representative class of the volatile compounds. The highest value was detected in POC samples inoculated with YPA (89.21%) followed by BY (69.71%), A5y (66.54%), LI-60-17 (43.37%), WSC (42.60%), KI-30-1 (39.83%), and YAB (26.84%). In POC samples fermented by LAB (Figure 2 and Table S2), the highest concentration of alcohols was observed in sample inoculated with BC-T3-35 (58.81%) followed by K-1 (46.35%), C-34 (45.34%), and C-11 (31.27%).

Isoamyl alcohols, benzyl-alcohol and phenyl-ethanol were the most abundant alcohols in almost all samples, with higher concentrations observed in POC samples fermented by yeasts. Phenyl-ethanol (2-PE) ranged from 6.04 to 27.47% (YAB and YPA, respectively) (Table S1). Lower values were observed in POC samples fermented by LAB (Table S2). In these samples the percentage of 2-PE ranged from 7.64% in C-11 to 15.06% in BC-T3-35. Isoamyl-alcohol was present in higher values in A5y (32.96%), and YPA (30.02%) while lower values were revealed in samples fermented by LAB (3.65–16.62%) (Table S2). 1-hexanol and 3-hexen-1-ol were detected in a few samples. In particular, 1-hexanol was identified in yeasts in only WSC and A5y strains, while 3-hexen-1-ol was found in BY, YPA and A5y strains. Alcohols have an important role in the production of esters, responsible for fruity notes. Among esters isoamyl acetate was the most representative in yeast-treated samples whereas ethyl octanoate was present in all LAB-treated ones. We also observed the presence of the sesquiterpene, farnesene, and furaneol. In POC fermented by yeasts, the total content of terpenes ranged from 5% in BY and 26.68% in YAB (Figure 1 and Table S1) while in bacteria fermented samples, we observed values ranging from 3.41% (C-34) to 16.84% (C-11) (Figure 2 and Table S2).

**Figure 3:**
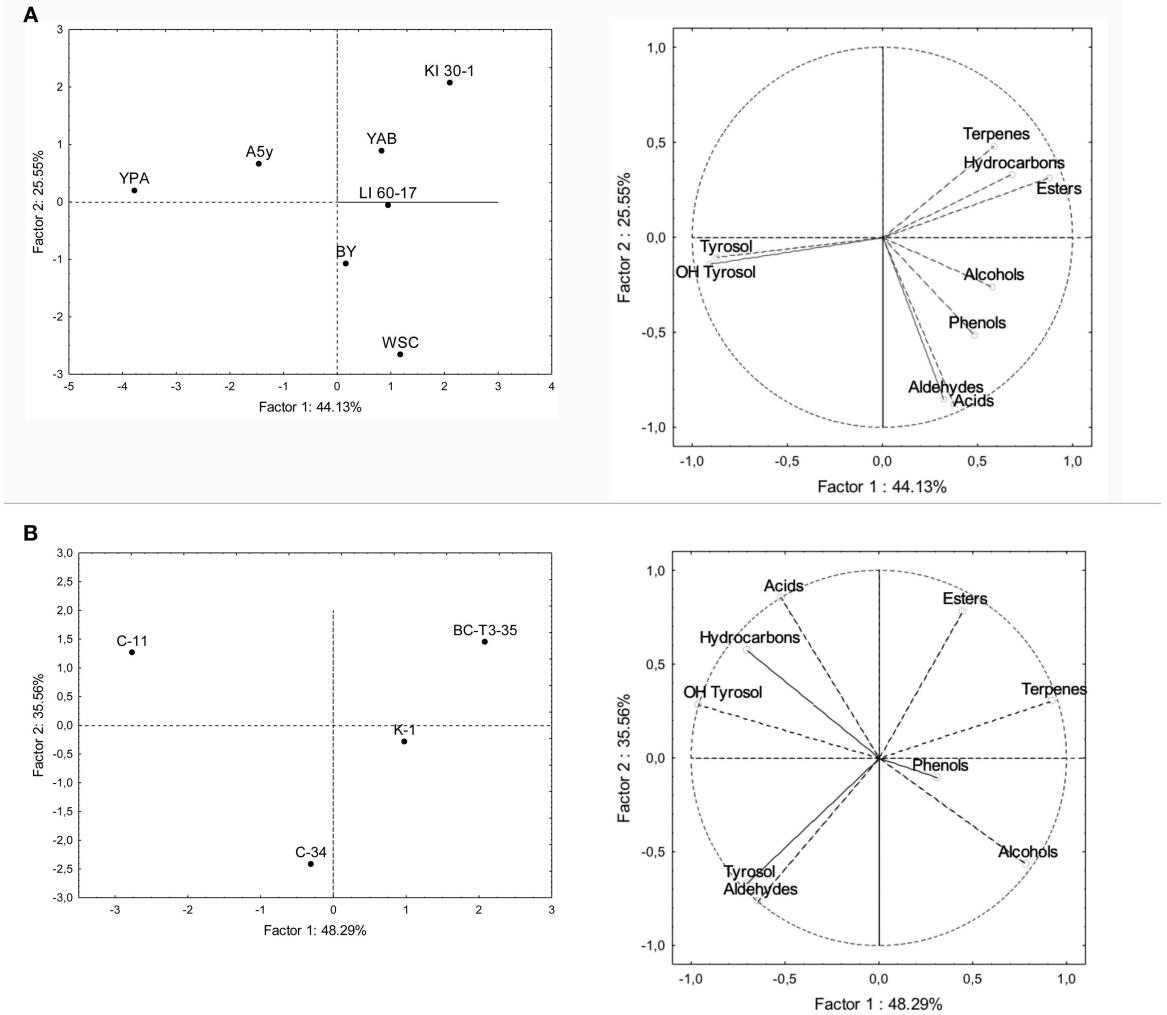
PCA of volatile compounds associated with **(A)** POC inoculated with yeast isolates and **(B)** POC inoculated with LAB isolates. PCA variables were the data obtained from the concentration and presence of volatile compounds and of main phenols and the different microbial isolates. The figure displays the sample scores and variable loadings in the planes formed by PC1–PC2.

Principal component analysis was applied to main phenolic compounds and to volatile classes produced at the end of the fermentations. In the bi-plot concerning fermented POC with different yeast strains, the total variance of the two main components was 69.68% (Figure 3A). PC2 separates terpens, hydroxycarbons and esters from phenols, alcohols, aldeydes and acids, while PC1 separates tyrosol and OH-tyrosol from the other components. The sample treated with the KI-30-1and YAB yeast isolates were located in the portion of the plane characterized by the presence of esters, terpenes and hydrocarbons (styrene). The sample inoculated with WSC isolate was associated with a greater production of acids (acetic acid) and aldehydes. The other samples treated with BY and LI-60-17 grouped together and volatile phenols and alcohols mainly characterize them. YPA and A5Y grouped in the plane mainly associated with tyrosol and hydroxytyrosol.

Samples treated with LAB showed a total variance of 83.85%, which is explained by two main components PC1 and PC2 (Figure 3B). The four samples were scattered in three main groups in the plane. The first one was represented by the C-34-inoculated sample which was associated with the presence of higher levels of tyrosol and aldehydes in the final product; the second one (C-11) was mainly characterized by a higher production of volatile acids, hydroxytyrosol and hydrocarbons, while BC-T3-35 and K-1 were positioned in the portion of the plane characterized by terpenes, phenols, alcohols and esters.

On the basis of the above reported results, yeast KI-30-1 and LAB BC-T3-35 isolates were selected as starters for pilot scale fermentations of POC.

### Pilot-Scale POC Fermentations

*Leccino* and *Cellina di Nardò* sterilized POC were inoculated with *Saccharomyces cerevisiae* ISPA-LE-KI 30-1 (KI-30-1) and *Leuconostoc mesenteroides* ISPA-LE-B-T3-35 (BC-T3-35). In both starter-driven fermentations, at the end of the process, yeasts were revealed throughout the process and their count corresponded to 1–3 × 10^5^ CFU/ml. *Enterobacteriaceae*, Staphylococci, coliforms, *Clostridia* was undetectable in starter-driven fermentations during the entire process (data not shown). After 30 days of yeast fermentation LAB isolate was inoculated and at the end of the process (50th day) LAB concentration resulted 5–7 × 10^6^ CFU/ml. pH values remained constant (4.8–4.9) within the first 30 days of fermentation and, after the 50th, they resulted 4.14 for POC from *Cellina di Nardò* and 4.21 for POC from *Leccino*. Glucose and fructose were almost totally consumed in both *Cellina di Nardò* and *Leccino* fermented POC samples, whereas sucrose was not detected in any of the samples. At the end of fermentation of the *Cellina* di *Nardò* POC the organic acid results were: lactic acid concentration was 7.51 ± 0.31 mg/g, tartaric acid 3.05 ± 0.13 mg/g, succinic acid 3.5 ± 0.16 mg/g; in *Leccino* fermented POC lactic acid concentration was 7.00 ± 0.26 mg/g, tartaric acid 2.25 ± 0.23 mg/g, malic acid 3.16 ± 0.14 mg/g, citric acid 4.02 ± 0.20 mg/g.

### Analysis of Volatile Compounds in POC Fermented by Selected Yeast and LAB Strains

Volatile compounds were analyzed in *Cellina di Nardò* and *Leccino* fermented POC, using the SPME-GC/MS technique (Table 1). When POC samples were fermented, firstly by yeast starter KI-30-1 and then by LAB isolate BC-T3-35, volatile profiles were significantly modified in comparison to non-fermented samples (NF). The volatile profile was characterized by high concentrations of alcohols, esters, terpenes and acids followed by minor compounds such as sulfur compounds, norisoprenoids, hydrocarbons, volatile phenols and aldehydes. Yeast metabolic activities induced a statistically increase of some volatile classes such as alcohols, esters, terpenes, hydrocarbons (*p* < 0.05). In *Cellina* di *Nardò* POC yeast fermented sample, showed a significant increase of volatile acids (*p* = 0.026) and a decrease of aldehydes (*p* = 0.013), whereas in *Leccino* POC samples treated with yeast there was a no significant change in aldehydes content and acids were not detectable. Among alcohols, 2-methyl propanol, isoamyl alcohols, and phenylethanol increased.

**Table 1:** Volatile compounds associated with Cellina di Nardò and Leccino Patè Olive cake (POC) unfermented (NF), fermented by selected yeast strain KI-30-1 (FY, 30 days) and fermented by yeast (FY, KI-30-1) and LAB (BC-T3-35) selected strains (FYL, 50 days).

**Volatile compounds**	**Cellina di Nardò POC**			**Leccino POC**		
	**NF**	**FY**	**FYL**	**NF**	**FY**	**FYL**
	**μg/Kg ± sd**	**μg/Kg ± sd**	**μg/Kg ± sd**	**μg/Kg ± sd**	**μg/Kg ± sd**	**μg/Kg ± sd**
**ESTERS**						
Ethyl acetate	0.95 ± 0.24^a^	1.58 ± 0.44^a^	2.76 ± 0.44^b^	0.68 ± 0.16^a^	3.87 ± 0.76^b^	4.56 ± 0.94^b^
Isomyl acetate	0.55 ± 0.11^a^	1.38 ± 0.16^b^	2.25 ± 0.36^c^	nd	2.67 ± 0.56 ^a^	3.82 ± 0.44^a^
Methyl Hexanoate	nd	nd	0.55 ± 0.11	nd	0.15 ± 0.005^a^	0.22 ± 0.07^a^
Ethyl hexanoate	nd	3.87 ± 0.94^a^	5.65 ± 1.56^a^	nd	0.56 ± 0.08^a^	0.71 ± 0.23^a^
Hexyl acetate	nd	2.10 ± 0.56^a^	2.29 ± 0.43^a^	nd	0.18 ± 0.006^a^	0.27 ± 0.06^b^
Methyl octanoate	nd	nd	2.90 ± 0.55	nd	1.67 ± 0.07 ^a^	2.70 ± 0.54^b^
Ethyl octanoate	2.10 ± 0.43^a^	6.18 ± 1.74^ab^	10.57 ± 2.90^b^	1.94 ± 0.34^a^	5.37 ± 0.76^ab^	7.95 ± 2.33^b^
Methyl decanoate	nd	nd	0.31 ± 0.06	nd	nd	0.34 ± 0.06
Methyl 2 furoate	nd	3.26 ± 0.85^a^	4.32 ± 0.54^a^	1.95 ± 0.26^a^	3.44 ± 0.73^ab^	4.34 ± 0.74^b^
Ethyl decanoate	nd	2.96 ± 0.43^a^	4.86 ± 0.65^b^	nd	0.25 ± 0.07^a^	0.41 ± 0.06^b^
Methyl salycilate	nd	3.06 ± 0.76^a^	6.13 ± 2.56^b^	nd	2.44 ± 0.48^a^	3.80 ± 0.84^a^
Phenyl acetate	0.87 ± 0.13^a^	1.89 ± 0.47^b^	2.80 ± 0.44^b^	nd	1.95 ± 0.11^a^	2.27 ± 0.54^a^
Total	4.47 ± 0.91	26.68 ± 6.35	5.39 ± 10.60	4.57 ± 0.76	22.55 ± 3.63	26.83 ± 5.91
**ALCOHOLS**						
2 Methylpropanol	0.71 ± 0.11^a^	3.97 ± 0.66^b^	4.17 ± 0.15^b^	1.20 ± 0.07^a^	3.77 ± 0.85^b^	4.17 ± 0.85^b^
Isoamylalcohols	nd	19.55 ± 4.17^a^	28.4 ± 5.10^a^	nd	20.67 ± 5.15^a^	27.64 ± 4.77^a^
Hexanol	nd	0.85 ± 0.14^a^	1.71 ± 0.16^a^	nd	nd	1.60 ± 0.45
3 Hexenol (Z)	2.10 ± 0.05^b^	1.87 ± 0.35^b^	1.12 ± 0.06^a^	3.06 ± 0.65^b^	2.93 ± 0.55^b^	2.16 ± 0.46^a^
1 Octen 3 ol	3.10 ± 0.44^b^	1.84 ± 0.37^a^	1.02 ± 0.06^a^	3.78 ± 0.78^b^	1.77 ± 0.54^a^	1.23 ± 0.26^a^
1 Heptanol	nd	nd	2.94 ± 0.46	nd	1.16 ± 0.05^a^	2.99 ± 0.75^b^
1 nonanol	nd	nd	1.77 ± 0.32	nd	nd	3.10 ± 0.55
Benzylalcohol	nd	nd	3.40 ± 0.48	nd	nd	2.94 ± 0.65
Phenylethanol	3.10 ± 0.93^a^	17.05 ± 3.67^b^	20.46 ± 4.06^b^	nd	8.37 ± 2.15^a^	10.35 ± 2.64^a^
Total	9.01 ± 1.53	45.13 ± 9.36	64.99 ± 10.85	8.04 ± 1.50	38.67 ± 9.29	56.18 ± 11.38
**ALDEHYDES**						
2 Heptanone	nd	nd	0.22 ± 0.05	nd	nd	nd
2 Hexenal	3.17 ± 0.44^b^	0.56 ± 0.15^a^	nd	nd	nd	nd
Octanal	0.78 ± 0.25^b^	0.74 ± 0.18^b^	0.34 ± 0.05^a^	0.88 ± 0.20^b^	0.38 ± 0.12^a^	0.25 ± 0.07^a^
*cis* 2-Decenal	nd	nd	nd	0.95 ± 0.25^b^	0.46 ± 0.08^a^	0.34 ± 0.05^a^
Furfural	0.44 ± 0.06^a^	1.28 ± 0.05^b^	1.45 ± 0.15^b^	nd	nd	nd
2 Octanone	3.10 ± 0.45^b^	2.67 ± 0.46^b^	0.32 ± 0.06^a^	nd	2.26 ± 0.43^a^	3.62 ± 0.47^b^
Octanal	3.20 ± 0.55^b^	0.56 ± 0.07^a^	0.17 ± 0.04^a^	2.86 ± 0.95^c^	1.55 ± 0.63^b^	0.21 ± 0.06^a^
Nonanal	3.86 ± 0.48^b^	2.15 ± 0.07^a^	1.76 ± 0.16^a^	3.10 ± 0.76^b^	0.34 ± 0.07^a^	nd
Decanal	4.28 ± 1.15^b^	2.94 ± 0.45^b^	1.06 ± 0.04^a^	3.76 ± 0.56^c^	1.93 ± 0.35^b^	0.96 ± 0.06^a^
Benzaldehyde	4.11 ± 0.83^c^	2.50 ± 0.36^b^	0.81 ± 0.23^a^	3.93 ± 0.65^a^	7.12 ± 2.84^b^	10.82 ± 2.65^c^
*trans* 2-Decenal	3.66 ± 0.46^c^	1.94 ± 0.28^b^	0.86 ± 0.34^a^	3.54 ± 0.94^c^	2.77 ± 0.73^b^	1.38 ± 0.04^a^
Cinnamaldehyde	0.35 ± 0.07^a^	0.68 ± 0.32^b^	0.81 ± 0.27^b^	nd	nd	1.25 ± 0.12
Total	26.95 ± 4.74	16.02 ± 2.39	7.80 ± 1.39	19.02 ± 4.31	16.81 ± 5.25	18.83 ± 3.52
**TERPENES**						
Citrale	0.06 ± 0.03^a^	0.38 ± 0.06^b^	0.59 ± 0.16^b^	nd	nd	3.12 ± 0.55
Limonene	0.16 ± 0.05^a^	0.40 ± 0.06^b^	0.49 ± 0.17^b^	0.14 ± 0.05^a^	0.22 ± 0.05^a^	0.30 ± 0.07^b^
*Trans*-β-ocimene	0.95 ± 0.28^a^	3.90 ± 0.45^b^	4.13 ± 0.65^b^	0.87 ± 0.15^a^	3.56 ± 0.84^b^	4.16 ± 0.64^b^
Farnesene	0.37 ± 0.15^a^	0.77 ± 0.14^b^	0.87 ± 0.43^b^	nd	nd	0.46 ± 0.15
*Cis* linalolox	nd	nd	0.41 ± 0.11	nd	nd	nd
Linalol	0.76 ± 0.23^a^	1.83 ± 0.17^b^	2.37 ± 0.43^b^	nd	nd	nd
Total	2.30 ± 0.74	7.28 ± 0.88	8.86 ± 1.95	1.01 ± 0.20	3.78 ± 0.89	8.04 ± 1.41
**HYDROCARBONS**						
Styrene	0.66 ± 0.15^a^	2.98 ± 0.94^b^	3.055 ± 1.05^b^	1.20 ± 0.16^a^	2.34 ± 0.66^b^	2.60 ± 0.25^b^
**SULFUR COMPOUNDS**						
Dimethyl sulfide	0.55 ± 0.11^b^	0.32 ± 0.08^a^	0.24 ± 0.07^a^	0.76 ± 0.12^b^	0.67 ± 0.15^b^	0.43 ± 0.12^a^
Methionol	nd	0.55 ± 0.10^a^	0.74 ± 0.22^a^	nd	nd	0.70 ± 0.22
Total	0.55 ± 0.11	0.87 ± 0.18	0.98 ± 0.29	0.76 ± 0.12	0.67 ± 0.15	1.13 ± 0.35
**NORISOPRENOIDS**						
β-damascenon	0.66 ± 0.004^a^	1.56 ± 0.23^b^	1.70 ± 0.17^b^	0.054 ± 0.007^a^	0.58 ± 0.08^b^	0.60 ± 0.16^b^
**VOLATILE PHENOLS**						
Guaiacol	nd	nd	0.085 ± 0.015	nd	nd	0.50 ± 0.15
**ACIDS**						
Acetic acid	nd	nd	4.21 ± 0.45	nd	nd	2.10 ± 0.44
Hexanoic acid	0.76 ± 0.25^a^	2.17 ± 0.56^b^	2.89 ± 0.37^b^	nd	nd	3.21 ± 0.83
Octanoic acid	0.44 ± 0.15^a^	1.93 ± 0.77^b^	1.50 ± 0.15^b^	nd	nd	0.92 ± 0.15
Total	1.20 ± 0.40	4.10 ± 1.33	8.60 ± 0.97			6.23 ± 1.42

*Data is the mean of 3 replicate measurements ± standard deviation. nd, not detected. Within the same cultivar for each compound, different letters indicate significant differences within the same row (not fermented vs. fermented) (One-Way ANOVA, p < 0.05)*.

After fermentation by LAB isolate BC-T3-35, *Cellina* di *Nardò* POC sample revealed a statistically significant increase in esters (*p* = 0.039) and acids (*p* = 0.003) classes in comparison to the same sample fermented only by yeast, whereas aldehydes were further reduced (*p* = 0.044). In the corresponding sample of *Leccino*, LAB metabolism was responsible of a significant increase of terpens (*p* = 0.004) and of the appearance of acids (when compared to the same sample treated with yeast).

Ester production increased during fermentation reaching a total level (at the end of the process) of 26.83 μg/kg in *Leccino* and 45.39 μg/kg in *Cellina di Nardò*. In *Leccino* fermented POC, ethyl acetate, ethyl octanoate and methyl 2 furoate were the major representative species of ester class, while in *Cellina di Nardò* fermented POC, the most abundant esters were ethyl hexanoate, ethyl octanoate, ethyl decanoate, methyl 2 furoate and methyl salycilate. As far as aldehydes, benzaldehyde was the major compound in fermented *Leccino* POCs, whereas the *Cellina di Nardò* sample contained higher a concentration of furfural, non-anal and decanal. Different terpenes were identified in the fermented POC, in particular in *Cellina di Nardò*. The total amount of terpenes increased from 2.30 μg/kg to 8.86 μg/kg, while in fermented Leccino POC the total amount of terpenes increased from 1.01 to 8.04 μg/kg.

### Chemical Characterization of POC Principal Nutritional Profile Constituents

Table 2 shows the chemical characterization of the principal constituents of the unfermented or fermented *Leccino* and *Cellina di Nardò* POC samples. The POC is characterized by the presence of several bioactive compounds such as polyphenols, triterpenic acids, tocochromanols, and carotenoids. The fermentation processes modified the bioactive phenols. A reduction of verbascoside and Oleacin (3,4-DHPEA-EDA) and a corresponding increase of hydroxytyrosol, mainly derived from the hydrolytic activities of lactic acid bacteria, were revealed in both the cultivar studied. Maslinic acid was higher than oleanoic acid, in all samples. Maslinic acid content was about 1.16 ± 0.05 mg/g FW, oleanoic acid ranged from 0.32 ± 0.04 mg/g FW (*Leccino*) to 0.43 ± 0.07 mg/g FW (*Cellina di Nardò*), these values were not significantly different (*p* > 0.05) when compared with the corresponding fermented POC samples.

**Table 2:** Chemical composition of the main bioactive compounds (polyphenols, triterpenic acids, tocochromanols, and carotenoids) and fatty acids profile of Patè olive cake (POC) from the cultivar *Leccino* and *Cellina di Nardò*, before and after the fermentation process.

	**Leccino POC**			**Cellina di Nardò POC**		
	**Not fermented**	**Fermented**	***P*-value^**Ω**^**	**Not fermented**	**Fermented**	***P*-value^**Ω**^**
**POLYPHENOLS (mg/g FW)**						
Hydroxytyrosol (3,4-DHPEA)	1.8 ± 0.1^b^	2.5 ± 0.1^a^	0.001	6.2 ± 0.2^b^	8.8 ± 0.5^a^	0.001
Tyrosol (p-HPEA)	0.7 ± 0.06^a^	0.6 ± 0.04^a^	0.074	0.8 ± 0.02^a^	0.8 ± 0.05^a^	1
Vanillic acid	0.8 ± 0.02^a^	0.2 ± 0.01^b^	< 0.001	0.5 ± 0.02^a^	0.3 ± 0.02^b^	< 0.001
hydroxytyrosol acetate	0.7 ± 0.01^a^	0.5 ± 0.01^b^	< 0.001	0.5 ± 0.0001^a^	0.5 ± 0.01^a^	1
*p*-coumaric acid	0.3 ± 0.02^a^	0.3 ± 0.01^a^	1	0.2 ± 0.01^a^	0.15 ± 0.01^b^	0.004
Verbascoside	0.7 ± 0.03^a^	0.6 ± 0.01^b^	0.006	3.7 ± 0.18^a^	1.9 ± 0.05^b^	< 0.001
Isoverbascoside	0.6 ± 0.04^a^	0.5 ± 0.01^b^	0.014	0.4 ± 0.01^a^	0.3 ± 0.02^b^	0.002
Oleacein (3,4-DHPEA-EDA)	1.4 ± 0.09^a^	0.4 ± 0.03^b^	< 0.001	5.0 ± 0.2^a^	1.9 ± 0.1^b^	< 0.001
Oleochantal P-HPEA-EDA	0.15 ± 0.01^a^	0.10 ± 0.01^b^	0.004	0.4 ± 0.001^a^	0.2 ± 0.001^b^	< 0.001
Rutin	0.002 ± 0.001^a^	0.001 ± 0.002^a^	0.482	0.06 ± 0.001^a^	0.05 ± 0.001^b^	< 0.001
(+)-Pinoresinol	nd	nd		0.3 ± 0.1^a^	0.3 ± 0.1^a^	1
Total	7.1 ± 0.4^a^	5.7 ± 0.2^b^	0.04	18.1 ± 0.7^a^	15.2 ± 0.9^b^	0.012
**TRITERPENIC ACIDS (mg/g FW)**						
Maslinic acid	1.16 ± 0.05^a^	1.27 ± 0.12^a^	0.217	1.16 ± 0.15^a^	1.31 ± 0.09^a^	0.212
Oleanoic acid	0.32 ± 0.04^b^	0.48 ± 0.09^a^	0.048	0.43 ± 0.07^a^	0.56 ± 0.08^a^	0.102
Total	1.48 ± 0.09^*a*^	1.74 ± 0.21^*a*^	0.12	1.59 ± 0.21^*a*^	1.87 ± 0.17^*a*^	0.147
**TOCOCHROMANOLS (μg/g FW)**						
α-T	23.20 ± 0.70^a^	21.10 ± 1.20^a^	0.059	21.90 ± 0.92^a^	22.01 ± 0.51^a^	0.865
ß-T3	1.74 ± 0.11^a^	1.54 ± 0.08^a^	0.064	1.25 ± 0.20^a^	1.37 ± 0.14^a^	0.443
Total	24.94 ± 0.81^a^	22.64 ± 1.28^a^	0.058	23.15 ± 1.12^a^	23.30 ± 0.65^a^	0.851
**CAROTENOIDS (μg/g FW)**						
Lutein	2.21 ± 0.31^a^	1.89 ± 0.02^a^	0.149	1.97 ± 0.11^a^	2.20 ± 0.30^a^	0.281
Zeaxanthin	0.06 ± 0.01^a^	0.07 ± 0.01^a^	0.288	0.05 ± 0.01^a^	0.07 ± 0.01^a^	0.07
α-Carotene	0.25 ± 0.05^a^	0.35 ± 0.06^a^	0.091	0.26 ± 0.01^a^	0.28 ± 0.10^a^	0.748
β-Carotene	0.74 ± 0.14^a^	0.55 ± 0.01^a^	0.079	0.84 ± 0.04^a^	0.84 ± 0.07^a^	1
13 cis β-Carotene	0.32 ± 0.06^a^	0.40 ± 0.07^a^	0.207	0.32 ± 0.02^a^	0.39 ± 0.05^a^	0.053
Total	3.58 ± 0.57^a^	3.26 ± 0.17^a^	0.404	3.44 ± 0.19^a^	3.78 ± 0.53^a^	0.305
**FATTY ACIDS (%)**						
Palmitic (C16:0)	17.94 ± 0.69^a^	17.23 ± 0.65^a^	0.264	16.73 ± 0.59^b^	18.40 ± 0.43^a^	0.017
Palmitoleic (C16:1)	1.82 ± 0.08^b^	2.08 ± 0.04^a^	0.007	1.86 ± 0.06^b^	2.33 ± 0.01^a^	< 0.001
Stearic (C18:0)	2.79 ± 0.02^b^	5.12 ± 0.89^a^	0.011	3.05 ± 0.07^b^	3.78 ± 0.05^a^	< 0.001
Oleic (C18:1 n−9)	63.01 ± 0.05^a^	62.46 ± 1.2^a^	0.472	63.11 ± 0.44^a^	62.11 ± 1.21^a^	0.250
Linoleic (C18:2 n−6)	13.62 ± 0.02^a^	13.11 ± 0.11^b^	0.002	14.47 ± 0.14^a^	13.38 ± 0.32^b^	0.006
Linolenic (C18:3 n−3)	0.91 ± 0.01	nd		0.78 ± 0.31	nd	
SFA	20.73 ± 0.71^a^	22.35 ± 1.54^a^	0.173	19.78 ± 0.66^b^	22.18 ± 0.48^a^	0.007
MUFA	64.83 ± 0.13^a^	64.54 ± 1.24^a^	0.708	64.97 ± 0.50^a^	64.44 ± 1.22^a^	0.525
PUFA	14.53 ± 0.03^a^	13.11 ± 0.11^b^	< 0.001	15.25 ± 0.45^a^	13.38 ± 0.32^b^	0.004
*PUFA/SFA*	*0.71*	*0.59*		*0.77*	*0.60*	

*The data represents the mean ± standard deviation of three replicate measurements (n = 3). nd, undetected; FW, fresh weight. Within the same cultivar for each compound, different letters indicate significant differences (^**Ω**^Student's test, p < 0.05) within the same row (not fermented vs. fermented)*.

The content of tocochromanols (tocopherols and tocotrienols) and carotenoids did not change significantly (*p* > 0.05) after the fermentation process. Among tocochromanols, α-tocopherol (α-T) and ß-tocotrienol (ß-T3) forms were detected. α-T content ranged from 21.10 ± 1.20 μg/g FW to 23.20 ± 0.65 μg/g FW. In turn, ß-T3 showed a minor tocochromanol component ranging from 1.25 ± 0.20 μg/g FW to 1.74 ± 0.11 μg/g FW).

Carotenoids were detected in small quantities, lutein was the most abundant (from 1.97 ± 0.11 μg/g FW to 2.21 ± 0.31 μg/g FW), followed by ß-carotene (from 0.55 ± 0.01 μg/g FW to 0.84 ± 0.04 μg/g FW), α-carotene (from 0.25 ± 0.05 μg/g FW to 0.35 ± 0.06 μg/g FW), 13 *cis* ß-carotene (from 0.32 ± 0.06 μg/g FW to 0.40 ± 0.07 μg/g FW) and zeaxanthin (from 0.05 ± 0.01 μg/g FW to 0.07 ± 0.01 μg/g FW).

The fatty acid profiles were characterized in both unfermented and fermented POC samples. Results showed that mono-unsaturated fatty acid (MUFA) was about 65% of total fatty acids, saturated fatty acids (SFA) was about 20% and polyunsaturated fatty acids (PUFA) were about 14%. As expected, the most abundant fatty acid was oleic acid followed by palmitic, linoleic and stearic acids. Other fatty acids were found in small quantities. In comparison with the unfermented POCs, the total SFA resulted in higher levels and PUFA showed lower levels, while the level of MUFAs remained unchanged.

The analyses of main nutritional parameters (fibers, fats content, humidity, ashes, total carbohydrates, total nitrogen, etc.) of both non-fermented and fermented POC samples were reported in Table S3.

## Discussion

POC, obtained by the two-phase centrifugal system, is thought to be a worthy by-product for further investigation. It offers a valuable resource for the formulation of new food, food supplements and feed considering its interesting compositional traits (19, 21). In order to verify its possible use in human food preparations a wide characterization of chemical composition in terms of main bioactive compounds and fatty acids was carried out. Moreover, in this study fermentation by selected microorganisms was used in order to produce a new valuable product from POC. The idea was to use already characterized yeast and LAB isolates able to survive and grow in hard niches such as wine, beer, olive mill wastewaters, and table brines characterized by the presence of difficult constraints, i.e., polyphenols, salt, alcohols, etc. On yeasts, one commercially available wine starter, two beer starters, one baker's starter together with two selected isolates for table olive preparation and an isolate able to detoxify OMW were tested. LAB isolates were all derived from fermented table olives.

In the first part of this study, the metabolic activity of each yeast and LAB isolates in the presence of 50% lyophilized and sterilized POC were tested. Beside sugar consumption and organic acid evolution during fermentation, the levels of the main and healthy important phenolic compounds were followed. The level of the highly valuable hydroxytyrosol increased in fermented POC samples. This was most likely due to microbial glucosidase and esterase activity (43, 44). Volatile compounds produced after fermentation by each isolate were studied. All compounds were identified in POC samples, i.e., alcohols, esters, hydrocarbons, terpenes, acids, and volatile phenols, were already described as associated with table olives *Cellina di Nardò* and *Leccino* (23), *Kalamàta* and *Conservolea* (24), *Nocellara del Belice* (45), *Manzanilla, Gordal*, and *Hojiblanca* (46), “alcaparras stone” from Portugal (47).

The PCA analysis, carried out on polyphenols and volatile compounds identified in POC samples inoculated with different yeasts and LAB isolates, gave the possibility to select KI-30-1 and BC-T3-35 isolates as the more promising microorganisms as candidate starters to be used in pilot-scale fermentation. KI-30-1 and BC-T3-35 isolates were able to develop main important volatile classes (i.e., esters and terpens) as well as to produce high levels of tyrosol and hydroxytyrosol in POC fermented samples. For these reasons these two isolates were chosen for pilot-scale experiments.

In pilot-scale fermentations, a sequential inoculation strategy was adopted. *Leccino* and *Cellina di Nardò* POC samples were first inoculated with the strain *S. cerevisiae* KI-30-1 and then with the strain *L. mesenteroides* BC-T3-35. This strategy has already been described on table olive fermentation, where yeasts were demonstrated to play a substantial role throughout the process and LAB, carried out lactic fermentation in the last part of fermentation (23–25). The trends of sugar consumption and the evolution of organic acids (lactic, citric, tartaric and acetic acid) in all starter-inoculated fermentations were in accordance with results reported for green and black olive fermentations (23–25, 48–50). In this work, the use of starters enabled the decrease of pH value in all fermented samples to about 4.2, which is adequate for fermented products such as black olives (25, 50). Beside the low pH value, the safety of the process and the final products were ensured by the ascertained absence of microbial contaminants. In POC samples yeast metabolic activities induced an increase in some volatile molecules such as alcohols, terpenes, hydrocarbons and volatile acids compared with un-inoculated samples. With regards to alcohols, 2-phenyl-ethanol (2-PE) is the aromatic alcohol, responsible for rose notes, produced from L-phenylalanine (51). Yeast species are responsible for the production of 2-PE and isoamyl-alcohols, the most representative molecules of alcohol class, thus these compounds can be indicative of yeast metabolism (25, 51, 52). The production of higher alcohols (isoamyl alcohols) is linked to microbial catabolism of amino acids (53). Higher alcohols are secondary products of alcoholic fermentation driven by yeasts, and can be influence with positive and negative notes, the aroma and flavor of fermented food. Higher concentrations of higher alcohols can produce a strong, pungent smell and taste whereas optimal levels can be responsible of fruity notes (54, 55). Formation of ethyl esters is mainly ascribable to yeast fermentation and to ethanolysis of acylCoA derived from fatty acid synthesis or degradation (56). Both esters and acetates can positively influence aroma by their characteristic grape-like odor, sweet-fruity and sweet-balsamic notes (52, 57). The key representative species of ester in fermented POCs were ethyl acetate, ethyl decanoate, ethyl hexanoate, ethyl octanoate, and methyl 2 furoate. The presence of these compounds has also been reported in table olives (25, 58) as well as in other fermented products (59, 60). Very interestingly, the methyl salycilate, revealed in yeast and LAB fermented *Cellina di Nardò* POC, has already been reported as an important volatile flavor compound to differentiate semi- and fully-fermented teas (61, 62). Concerning aldehydes, according to Malheiro et al. (63), these compounds are present in high concentrations in all studied olive cultivars. In green olives aldehyde content can extend to 50% of all volatile classes and even 75% in black olives (64). Terpenes and norisoprenoids are generally present as glycosylated precursors and can be released by enzymatic hydrolysis during fermentation (24, 65). The increase of terpenes and norisoprenoids in fermented POC could be ascribable to glycosidase activities of inoculated microbial starters.

POCs, either fermented or not, represent a good source of triterpenic acids. Several studies have indicated that maslinic and oleanoic acids have anti-inflammatory, antitumoral, antihyperglicemic, hepatoprotective, cardioprotective, and antimicrobial effects (66). These bioactives are also of interest to food and cosmetic industries (14, 67). Maslinic and oleanoic acids are the main triterpenic acids found in table olive, olive oils and olive pomace oils (20, 40, 68). According to Padalino et al. (20), in POCs the content of maslinic acid were higher than oleanoic acid. Also the carotenoid profiles were similar to those obtained by Padalino et al. (20). Within tocochromanols, α-T is known to be the most biologically active, with a role in preventing lipid peroxidation and scavenging of lipid peroxyl radicals (69). α-T content observed in POCs was in agreement with the results found by Nunes et al. (14) in olive pomace from a two-phase extraction process.

The procedure for the production of fermented POC, based on sequential inoculums of a yeast LAB strains, allowed the standardization of the process and, obtaining a final product in a very limited period of time (50 days). The process can also be monitored following specific chemical descriptors suitable to describe the evolution of fermentative activities of yeasts and LAB, as already demonstrated for table olives (24, 25).

Experiments are now in progress to verify the possibility of further exploiting POC in the formulation of enriched food products (i.e., bakery products) as well as to characterize their nutritional and sensorial traits. Large-scale experiments are also now planned to improve and validate the use of these autochthonous starter cultures.

## Author Contributions

MT, MD, and GV conduction of the experiments, acquisition, and interpretation of the results. GB, MS, AT, and GM work design, data discussion, and manuscript preparation.

### Conflict of Interest Statement

The authors declare that the research was conducted in the absence of any commercial or financial relationships that could be construed as a potential conflict of interest.
