# Supplementary material for: Patè Olive Cake: Possible Exploitation of a By-Product for Food Applications
Source: Front Nutr. 2019 Feb 5;6:3. doi: 10.3389/fnut.2019.00003 (PMC6371699; doi:10.3389/fnut.2019.00003)
Supplement: Supplementary file 1 [file Table_1.DOCX]

Table S1: Volatile compounds of Patè Olive Cake fermented by different yeast strains. Each volatile compound was expressed as relative percentage of the peak area (%) on the total peak areas. nd, not detected.

|  |  |  | **Yeast strains** | |  |  |  |
| --- | --- | --- | --- | --- | --- | --- | --- |
| **Volatiles (%)** | **KI-30-1** | **A5y** | **LI-60-17** | **WSC** | **BY** | **YAB** | **YPA** |
| **Alcohols** |  |  |  |  |  |  |  |
| Isoamyl alcohols | 13.33 | 32.96 | 15.13 | 21.22 | 29.80 | 6.66 | 30.02 |
| 1-Hexanol | nd | 1.38 | nd | 5.04 | nd | nd | nd |
| trans 3-hexen-1-ol | nd | 1.88 | nd | nd | 24.99 | nd | 2.85 |
| Benzylalcohol | 15.27 | nd | 10.31 | 3.80 | 8.01 | 14.14 | 8.35 |
| Isopropanol | nd | 16.79 | nd | nd | nd | nd | 20.51 |
| Phenylethanol | 11.22 | 13.53 | 17.93 | 12.54 | 6.90 | 6.04 | 27.47 |
| **Total** | **39.83** | **66.54** | **43.37** | **42.60** | **69.71** | **26.84** | **89.21** |
|  |  |  |  |  |  |  |  |
| **Esters** |  |  |  |  |  |  |  |
| Isoamyl acetate | 8.98 | 13.21 | 13.66 | 10.54 | 6.55 | 19.94 | nd |
| Ethyl octanoate | 7.96 | nd | 3.35 | nd | 6.71 | nd | 3.14 |
| Ethyl decanoate | 2.51 | nd | nd | nd | nd | nd | nd |
| **Total** | **19.45** | **13.21** | **17.01** | **10.54** | **13.25** | **19.94** | **3.14** |
|  |  |  |  |  |  |  |  |
| **Terpenes** |  |  |  |  |  |  |  |
| Farnesene | 7.72 | nd | 14.47 | nd | 5.01 | 26.68 | nd |
|  |  |  |  |  |  |  |  |
| **Hydrocarbons** |  |  |  |  |  |  |  |
| Styrene | 33.48 | 20.25 | 16.90 | 21.42 | nd | 13.00 | 7.65 |
|  |  |  |  |  |  |  |  |
| **Aldehydes** |  |  |  |  |  |  |  |
| Acetoin | nd | nd | nd | 3.27 | nd | nd | nd |
| Nonanal | nd | nd | nd | 15.78 | nd | nd | nd |
| Benzaldehyde | nd | nd | nd | nd | 7.13 | 6.50 | nd |
| **Total** |  |  |  | **19.05** | **7.13** | **6.50** |  |
|  |  |  |  |  |  |  |  |
| **Acids** |  |  |  |  |  |  |  |
| Acetic acid | nd | nd | 1.23 | 2.13 | 0.71 | nd | nd |
|  |  |  |  |  |  |  |  |
| **Phenols** |  |  |  |  |  |  |  |
| 4-ethylphenol | nd | nd | 7.02 | 4.27 | 4.19 | 7.04 | nd |
