# Supplementary material for: Patè Olive Cake: Possible Exploitation of a By-Product for Food Applications
Source: Front Nutr. 2019 Feb 5;6:3. doi: 10.3389/fnut.2019.00003 (PMC6371699; doi:10.3389/fnut.2019.00003)
Supplement: Supplementary file 2 [file Table_2.docx]

Table S2: Volatile compounds of Patè Olive Cake fermented by different lactic acid bacteria (LAB) strains. Each volatile compound was expressed as relative percentage of the peak area (%) on the total peak areas. nd, not detected.

| **LAB Strain** | | | | |
| --- | --- | --- | --- | --- |
| **Volatiles (%)** | **K-1** | **C-11** | **BC T3-35** | **C-34** |
| **Alcohols** |  |  |  |  |
| Isoamyl alcohols | 10.05 | 3.65 | 16.62 | 7.48 |
| 1-Hexanol | nd | 3.29 | 7.90 | 5.81 |
| trans 3-hexen-1-ol | 11.20 | 2.75 | nd | 6.08 |
| Benzylalcohol | 14.07 | 13.94 | 19.22 | 12.16 |
| Phenylethanol | 11.04 | 7.64 | 15.06 | 13.80 |
| **Total** | **46.35** | **31.27** | **58.81** | **45.34** |
|  |  |  |  |  |
| **Esters** |  |  |  |  |
| Isoamyl acetate | nd | nd | 6.96 | nd |
| Ethyl octanoate | 10.57 | 8.08 | 6.23 | 7.26 |
| **Total** | **10.57** | **8.08** | **13.19** | **7.26** |
|  |  |  |  |  |
| **Terpenes** |  |  |  |  |
| Farnesene | 4.58 | 9.28 | 5.71 | 3.41 |
| Furaneol | nd | 7.56 | 6.38 | nd |
| **Total** | **4.58** | **16.84** | **12.10** | **3.41** |
|  |  |  |  |  |
| **Hydrocarbons** |  |  |  |  |
| Styrene | 8.58 | 15.16 | 5.97 | 6.63 |
|  |  |  |  |  |
| **Aldehydes** |  |  |  |  |
| Nonanal | 7.53 | 9.17 | nd | 26.20 |
| Benzaldehyde | 8.25 | 13.94 | 4.05 | 5.15 |
| **Total** | **15.78** | **23.11** | **4.05** | **31.34** |
|  |  |  |  |  |
| **Acids** |  |  |  |  |
| Acetic acid | 1.96 | 5.53 | 5.88 | 1.69 |
|  |  |  |  |  |
| **Phenols** |  |  |  |  |
| Guaiacolo | 1.56 | nd | nd | nd |
| 4-ethylphenol | 10.31 | nd | nd | 4.32 |
| **Total** | **11.87** |  |  | **4.32** |
