# Supplementary material for: Patè Olive Cake: Possible Exploitation of a By-Product for Food Applications
Source: Front Nutr. 2019 Feb 5;6:3. doi: 10.3389/fnut.2019.00003 (PMC6371699; doi:10.3389/fnut.2019.00003)
Supplement: Supplementary file 3 [file Table_3.DOCX]

Table S3. Nutritional information of *Leccino* and *Cellina di Nardò* Patè olive cake (POC) ^*^

|  | | *Leccino* POC | |  |  | *Cellina di Nardò* POC | |
| --- | --- | --- | --- | --- | --- | --- | --- |
|  |  | Not fermented | Fermented |  |  | Not fermented | Fermented |
| Energy (Kcal/100g) | | 84.6±7.2 | 73.0±6.7 |  |  | 86.1±7.5 | 64.6±5.2 |
| Moisture (%) | | 78.3±6.2 | 91.6±8.6 |  |  | 79.3±5.1 | 91.1±6.8 |
| Ash (%) | | 1.4±0.01 | 1.5±0.02 |  |  | 1.2±0.1 | 1.4±0.1 |
| Nitrogenous substances (%) | | <0.50 | <0.50 |  |  | <0.50 | <0.50 |
| Carbohydrates (%) | | 11.4±1.6 | 10.2±1.2 |  |  | 9.2±0.9 | 6.4±0.6 |
| Total Dietary Fibres (%) | | 5.9±0.34 | 1.8±0.01 |  |  | 6.1±0.5 | 6.4±0.5 |
| Total fat (hydrolysis method) (%) | | 2.9±0.02 | 3.2±0.01 |  |  | 4.1±0.02 | 3.4±0.02 |

^*^Nutritional facts were determined by BonassisaLab SRL (Foggia, Italy) (http://www.bonassisa.it) adopting the procedure UNI CEI EN ISO/IEC 17025:2005.
